# Long-Term Outcomes in Survivors of Fulminant Myocarditis Supported with Venoarterial Extracorporeal Membrane Oxygenation by Discharge Cardiac Function

**DOI:** 10.1016/j.cjco.2026.03.020

**Published:** 2026-04-08

**Authors:** Maria Lambadaris, Julie K.K. Vishram-Nielsen, Takahiro Okumura, Yee-Chun Chen, Aristine Cheng, Hsin-Yun Sun, Antonio Loforte, Yasuhide Asaumi, Kenichiro Sawada, Mingjie Huang, Wei C. Lee, Thomas Fux, Matteo Pozzi, Heather J. Ross, Finn Gustafsson, Hasse Møller-Sørensen, Albert Ariza-Solé, Mario Senechal, Pierre Yves Turgeon, Manuel Martinez-Selles, Francisco J. Hernández-Pérez, Roberto Lorusso, Filio Billia, Ana C. Alba

**Affiliations:** a Peter Munk Cardiac Centre, University Health Network, Toronto, Ontario, Canada; b Department of Cardiology, Zealand University Hospital, Roskilde, Denmark; c Department of Cardiology, Nagoya University Graduate School of Medicine, Nagoya, Japan; d Division of Infectious Diseases, Department of Internal Medicine, National Taiwan University Hospital, Taipei, Taiwan; e Department of Cardiac Surgery, S. Orsola University Hospital, IRCCS Bologna, Bologna, Italy; f Department of Surgical Sciences, University of Turin, Turin, Italy; g Department of Cardiovascular Medicine, National Cerebral and Cardiovascular Center, Osaka, Japan; h Department of Cardiothoracic Surgery, National Heart Centre Singapore, Singapore, Singapore; i Division of Cardiology, Department of Internal Medicine, Kaohsiung Chang Gung Memorial Hospital, Chang Gung University College of Medicine, Kaohsiung, Taiwan; j Department of Perioperative Medicine and Intensive Care, Department of Physiology and Pharmacology, Karolinska Institute, Karolinska University Hospital, Stockholm, Sweden; k Department of Cardiac Surgery, Louis Pradel Cardiologic Hospital, Lyon, France; l Department of Cardiology, Rigshospitalet, University Hospital of Copenhagen, Copenhagen, Denmark; m Department of Clinical Medicine, University of Copenhagen, Copenhagen, Denmark; n Department of Cardiothoracic Anaesthesiology, Rigshospitalet, University Hospital of Copenhagen, Copenhagen, Denmark; o Department of Cardiology, Bellvitge University Hospital, L’Hospitalet de Llobregat, Barcelona, Spain; p Department of Cardiology, Quebec Heart and Lung Institute, Quebec City, Canada; q Cardiology Department, Hospital General Universitario Gregorio Marañón, Instituto de Investigación Sanitaria Gregorio Marañón, CIBERCV. School of Biomedical Sciences and Health, Universidad Europea de Madrid. School of Medicine, Universidad Complutense. Madrid, Spain; r Department of Cardiology, Heart Failure and Transplant Section, Puerta de Hierro Majadahonda University Hospital, Madrid, Spain; s Department of Cardio-Thoracic Surgery, Heart and Vascular Centre, Maastricht University Medical Centre, Maastricht, Cardiovascular Research Institute Maastricht, Maastricht, The Netherlands

**Keywords:** heart failure, extracorporeal membrane oxygenation, left ventricular ejection fraction, left ventricular assist device, heart transplantation

## Abstract

**Background:**

Fulminant myocarditis (FM) is a life-threatening cause of cardiogenic shock. Veno-arterial extracorporeal membrane oxygenation (VA-ECMO) is often employed as a bridge to recovery, but data on long-term outcomes and prognostic markers post-discharge remain limited. We described long-term outcomes and explored the association between discharge left ventricular ejection fraction (LVEF) and post-discharge events.

**Methods:**

This retrospective, international, multicentre cohort study included adult FM patients who required VA-ECMO and survived to hospital discharge. Patients were stratified by LVEF ≥ 50% or < 50% at discharge. Cox proportional hazards models were used to assess the association between discharge LVEF and the primary composite endpoint of death, heart transplantation, or durable mechanical circulatory support postdischarge.

**Results:**

A total of 106 patients from 11 centres were included, with a median age of 43 years (interquartile range 29-57); 42% were male. At discharge, LVEF was ≥ 50% in 69 patients and < 50% in 37 patients. Over a median follow-up of 4.4 years (interquartile range 1.6-7.9), 3 patients (2.8%) experienced the composite outcome (2 deaths, 1 left ventricular assist device implantation). Compared with patients discharged with an LVEF ≥ 50%, those with an LVEF < 50% had a numerically higher risk of the composite endpoint (hazard ratio 2.9, 95% confidence interval 0.19-42.9), although this difference was not statistically significant.

**Conclusions:**

Given the low number of postdischarge events, this study was underpowered to assess the prognostic value of discharge LVEF. However, long-term outcomes were favourable among survivors of FM supported with VA-ECMO, regardless of their LVEF at discharge.

## Central Illustration

**Figure:**
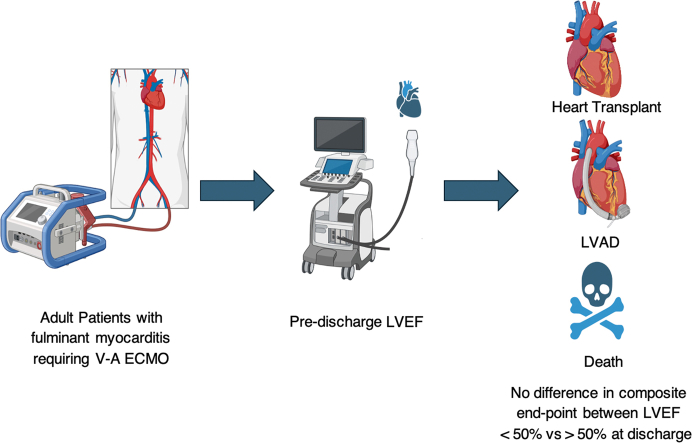

## Résumé

**Contexte:**

La myocardite fulminante (MF) est une cause de choc cardiogénique engageant le pronostic vital. L'oxygénation extracorporelle par membrane veino-artérielle (ECMO V-A) est souvent utilisée comme traitement de transition vers la récupération, mais les données sur les résultats à long terme et les marqueurs pronostiques après la sortie de l'hôpital restent limitées. Nous avons décrit les résultats cliniques à long terme et exploré l'association entre la fraction d'éjection ventriculaire gauche (FEVG) à la sortie et les événements survenus après la sortie.

**Méthodologie:**

Cette étude de cohorte rétrospective, internationale et multicentrique a inclus des patients adultes atteints de MF ayant nécessité une ECMO V-A et ayant survécu jusqu'à leur sortie de l'hôpital. Les patients ont été stratifiés selon une FEVG ≥ 50 % ou < 50 % à la sortie. Des modèles de risques proportionnels de Cox ont été utilisés pour évaluer l'association entre la FEVG à la sortie et le critère d'évaluation composite principal comprenant le décès, la transplantation cardiaque ou l'assistance circulatoire mécanique durable après la sortie.

**Résultats:**

Au total, 106 patients issus de 11 centres ont été inclus, avec un âge médian de 43 ans (intervalle interquartile [IIQ] 29 – 57); 42 % étaient des hommes. À la sortie, la FEVG était ≥ 50 % chez 69 patients et < 50 % chez 37 patients. Au cours d’un suivi médian de 4,4 (IIQ 1,6 – 7,9) ans, trois (2,8 %) patients ont présenté l’événement composite (2 décès, 1 assistance ventriculaire gauche). Par rapport aux patients sortis de l'hôpital avec une FEVG ≥ 50 %, ceux présentant une FEVG < 50 % avaient un risque numériquement plus élevé de survenue du critère composite (rapport des risques 2,9, intervalle de confiance à 95 % 0,19–42,9), bien que cela ne soit pas statistiquement significatif.

**Conclusions:**

Compte tenu du faible nombre d’événements après la sortie, cette étude n’avait pas la puissance statistique suffisante pour évaluer la valeur pronostique de la FEVG à la sortie. Cependant, les résultats à long terme étaient favorables chez les survivants d’une myocardite fulminante traités par ECMO V-A, indépendamment de la FEVG à la sortie.

Acute myocarditis is an inflammatory condition caused by autoimmune disorders or viral infections.^1^ The clinical presentation ranges widely from asymptomatic individuals to those who present with acute heart failure and cardiogenic shock, namely fulminant myocarditis (FM). FM is a rare but life-threatening condition characterized by rapid onset of severe biventricular dysfunction and hemodynamic collapse.^2^^,^^3^ Mechanical circulatory support (MCS), such as veno-arterial extracorporeal membrane oxygenation (VA-ECMO), can be used to support these patients.^4^ A recent systematic review demonstrated with moderate confidence that the short-term mortality incidence in patients with rapidly progressive FM requiring VA-ECMO was 35%, with a lower mortality incidence in younger patients.^5^

Given the potential for myocardial recovery in FM, assessment of cardiac function and its prognostic implication is an area of clinical interest. Left ventricular ejection fraction (LVEF) may serve as an indicator of myocardial recovery in both the short and long term. In a cohort of 99 patients with FM who were treated with VA-ECMO, a higher LVEF within 48 hours of cannulation was associated with increased chances of survival to hospital discharge without the need of durable MCS.^6^ Additionally, in a recent multicentre, Japanese registry with biopsy-proven FM in 214 patients, only 39% required ECMO support. At 1 year, those with an LVEF < 50% at the time of hospital discharge had a higher probability of death or heart transplantation (HTx), compared to those with an LVEF > 50% (4% vs 1%, *P* < 0.001).^7^ In addition to a small subset of patients supported with VA-ECMO, this study did not report on long-term prognosis among patients requiring VA-ECMO or on the impact on LVEF in this specific high-risk subpopulation. Thus, our multicentre international study aimed to describe long-term outcomes and explore the potential association between discharge LVEF and long-term outcomes in patients with FM supported with VA-ECMO who were discharged without any durable MCS or heart transplantation (HTx). The study design and outcomes are summarized in the Central Illustration.

**Central Illustration:**
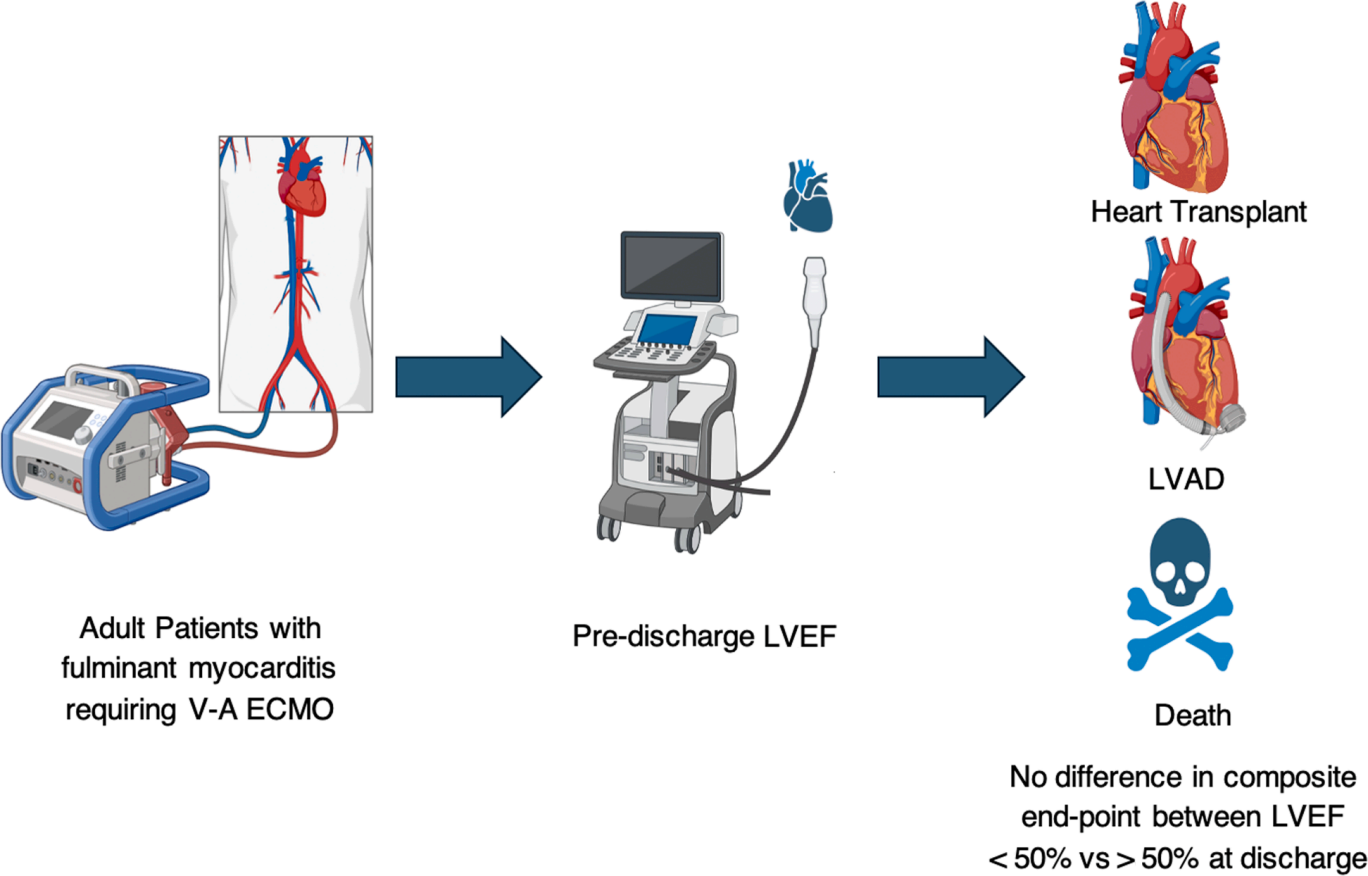
Outcomes after venoarterial extracorporeal membrane oxygenation **(**VA-ECMO) in fulminant myocarditis. This Central Illustration summarizes outcomes among adult patients with fulminant myocarditis who required VA ECMO support and survived to hospital discharge. Patients were stratified by left ventricular ejection fraction (LVEF) at discharge (≥ 50% vs < 50%). Cox proportional hazards analysis showed no statistically significant difference in the composite endpoint of death, heart transplantation, or left ventricular assist device (LVAD) implantation between groups, although those with LVEF < 50% had a higher but nonsignificant risk (hazard ratio 2.9, 95% confidence interval 0.19-42.9). These findings highlight hospital discharge as an important prognostic milestone irrespective of LVEF status.

## Materials and Methods

### Study design and patient population

**Figure 1:**
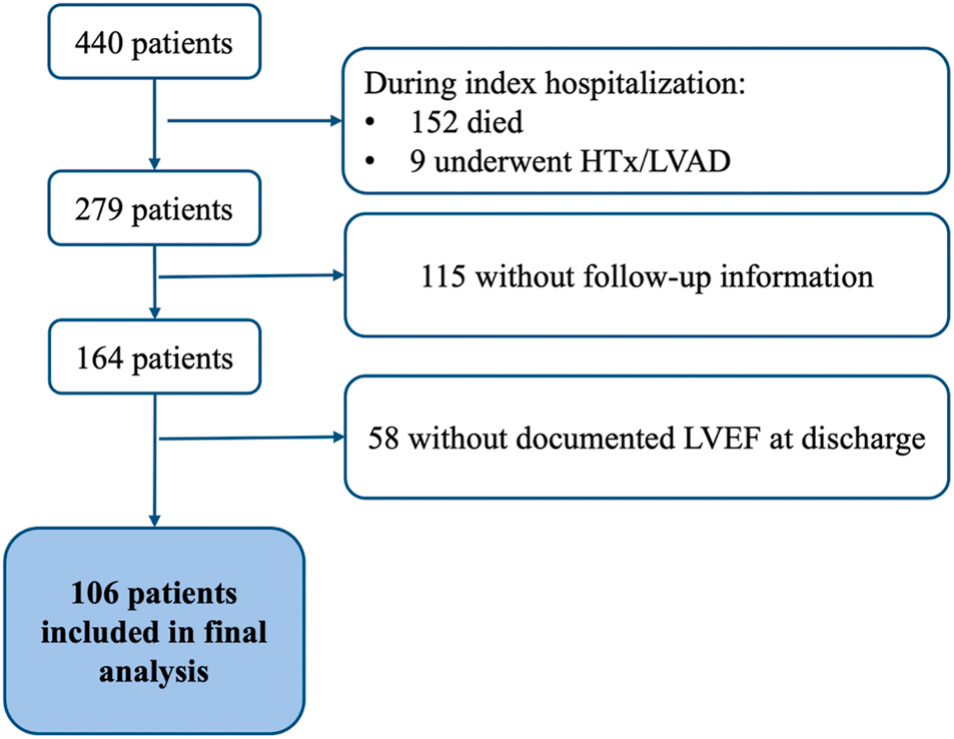
Patient selection. This flow diagram demonstrates the inclusion and exclusion criteria for the study cohort. HTx, heart transplantation; LVAD, left ventricular assist device; LVEF, left ventricular ejection fraction.

A recent systematic review published by our research lab identified observational cohort studies of adult FM patients that reported mortality after the initiation of VA-ECMO (13 studies on 871 patients with 383 short-term mortality events).^1^ The authors of each identified centre were contacted and invited to participate in the present individual patient–data meta-analysis. Sixteen centers agreed and provided data on patients with FM who were supported with VA-ECMO. FM diagnosis was established at each participating centre based on clinical presentation consistent with FM. Endomyocardial biopsy was performed when clinically indicated; in patients without biopsy confirmation, ischemic heart disease was excluded using coronary angiography or equivalent imaging, according to institutional practice. Diagnosis was supported by clinical features, laboratory biomarkers, and echocardiographic findings. Given the retrospective multicentre nature of the study, diagnostic criteria were not standardized across centres and included a spectrum of biopsy-proven and clinically diagnosed myocarditis cases. Of 440 patients with FM VA-ECMO, 152 died during the index hospitalization, and 9 underwent HTx or durable mechanical circulatory support during hospitalization, leaving 279 patients discharged without advanced therapy. Among these, 115 had no measurable post-discharge follow-up and were excluded. Of the remaining 164 patients, 58 lacked documented discharge LVEF and were excluded from the primary analysis. The final analytic cohort consisted of 106 patients from 11 centres (Fig. 1). After application of the inclusion and exclusion parameters, 11 centres contributed data for this study. To assess potential selection bias related to missing LVEF data, we compared baseline and management characteristics of patients with vs without available measures of discharge LVEF (Supplemental Table S1).

### Ethics

This work was approved by the respective ethics board at each participating hospital site.

### Clinical variables collected

Demographic and clinical information was extracted from the patient records at each centre and included the following: age; sex; race; height; weight; kidney function; lactate; presence of cardiac arrhythmias (ventricular fibrillation, ventricular tachycardia, complete heart block); use of mechanical ventilation; renal replacement therapy; in-hospital medications (inotropes, vasodilator, vasopressor); left ventricular venting (intra-aortic balloon pump, Impella, Johnson and Johnson, MA); use of immunosuppressive medications (steroids, intravenous immunoglobulin [IVIG]); and complications while on VA-ECMO (bleeding, sepsis, liver failure, limb ischemia, neurologic complications). LVEF was recorded at 3 predefined time points: prior to ECMO implantation, prior to ECMO decannulation, and prior to hospital discharge (defined as the last documented echocardiographic assessment before discharge).

### Classification of outcomes

During the post-discharge follow-up period, we collected outcomes including all-cause mortality, HTx, and need for left ventricular assist device (LVAD). The primary study endpoint was the composite of death, HTx, or LVAD.

### Statistical analysis

The study population characteristics were described using median and 25th and 75th percentiles for continuous variables. Categorical variables were presented as proportions. Patients were stratified by LVEF at hospital discharge using a 50% threshold, selected a priori based on prior contemporary studies in FM.^7^ We evaluated the association of LVEF at discharge with long-term mortality using univariable Cox proportional hazard models, reporting hazard ratios (HRs) and their 95% confidence intervals (CIs). Patients were followed from the time of hospital discharge until the occurrence of the composite endpoint (death, durable LVAD implantation, or HTx) or censored at the time of last known clinical follow-up. Follow-up information was obtained from participating centres using longitudinal outpatient records and institutional databases.

## Results

### Baseline characteristics

A total of 106 patients across 11 centres survived to hospital discharge without requiring HTx or durable LVAD support (Table 1). The median duration of VA-ECMO support, calculated from implantation to explantation, was 6 days (interquartile range 5-9 days). Baseline characteristics stratified by LVEF at hospital discharge (≥ 50% vs < 50%) are shown in Table 2. The median age was similar between groups; however, patients discharged with an LVEF < 50% were more frequently male, compared with those discharged with an LVEF ≥ 50%. A comparison between patients with available discharge LVEF and those without is presented in Supplemental Table S1. Patients with available LVEF differed in several management-related characteristics, including higher rates of mechanical ventilation, and immunomodulatory therapies such as steroids and IVIG. All observed postdischarge deaths occurred within the cohort with available discharge LVEF, whereas no post-discharge deaths were observed among patients without available LVEF data.

**Table 1:** Overall population stratified by centre

Centre	Overall population (N = 106)
Rigshospitalet, Copenhagen, Denmark	2 (1.9)
Karolinska Institute, Stockholm, Sweden	7 (6.6)
Louis Pradel Cardiologic Hospital, Lyon, France	8 (7.5)
Department of Cardiology, University of Turin, Turin, Italy	8 (7.5)
Bellvitge Hospital, Barcelona, Spain	4 (3.8)
Hospital Universitario Gregorio Marañón, Madrid, Spain	8 (7.5)
Toronto General Hospital, Toronto, Canada	29 (27.4)
Nagoya University Graduate School of Medicine, Nagoya, Japan	17 (16)
National Cerebral and Cardiovascular Centre, Suita, Japan	16 (15.1)
Institut Universitaire de Cardiologie et Pneumologie de Québec (Iucpq), Quebec, Canada	4 (3.8)
Hospital Universitario Puerta De Hierro Majadahonda, Madrid, Spain	2 (1.9)

Values are n (%).

**Table 2:** Baseline characteristics of the overall population

Variable	LVEF ≥ 50% (n = 69)	LVEF < 50% (n = 37)	*p*
Demographics			
Age, y	42.0 [31.0–53.0]	43.0 [28.0–60.0]	0.82
Male	23 (33.3)	21 (56.8)	0.024
BMI, kg/m^2^	22.9 [20.0–25.6]	22.8 [20.1–25.0]	1.00
Clinical parameters			
Creatinine, μmol/L	91.1 [73.0–131.7]	97.6 [79.6–131.8]	0.57
Cardiac arrhythmias			
Ventricular fibrillation	2 (2.9)	5 (13.5)	0.049
Ventricular tachycardia	6 (8.7)	8 (21.6)	0.075
Complete heart block	18 (26.1)	8 (21.6)	0.645
Left ventricular ejection fraction			
Pre-ECMO	20.0 [14.4–33.5]	20.1 [14.8–27.6]	0.37
Pre-decannulation	42.5 [33.4–50.0]	30.0 [24.6–40.8]	0.002
Pre-discharge	60.0 [57.5–66.2]	42.0 [30.6–47.0]	< 0.001
Management			
Mechanical ventilation	59 (85.5)	32 (86.5)	0.89
Renal replacement therapy	7 (10.1)	3 (8.1)	0.79
Inotropes	52 (75.4)	28 (75.7)	0.97
Vasodilators	47 (68.1)	24 (64.9)	0.74
Vasopressors	57 (82.6)	29 (78.4)	0.61
Left ventricular venting			
Intra-aortic balloon pump	46 (66.7)	25 (67.6)	0.93
Impella	2 (2.9)	1 (2.7)	1.00
Immunosuppressive therapies			
Steroids	23 (33.3)	12 (32.4)	0.92
Immunosuppressive therapy	5 (7.2)	2 (5.4)	0.71
IVIG	17 (24.6)	9 (24.3)	0.97
Complications on VA-ECMO			
Bleeding	26 (37.7)	14 (37.8)	0.99
Sepsis	13 (18.8)	7 (18.9)	0.99
Liver failure	35 (50.7)	19 (51.4)	0.94
Limb ischemia	7 (10.1)	4 (10.8)	0.91
Neurologic (ie, stroke)	4 (5.8)	2 (5.4)	1.00
ICU length of stay, d	14.0 [9.0–20.0]	15.0 [9.5–21.0]	0.63

Categorical variables are presented as n (%).Continuous variables are presented as median [first and third quartiles]. BMI, body mass index; ECMO, extracorporeal membrane oxygenation; ICU, intensive care unit; IVIG, intravenous immunoglobulin; LVEF, left ventricular ejection fraction; VA-ECMO, veno–arterial ECMO, Impella (Johnson and Johnson, MA).

Prior to ECMO cannulation, most patients required inotropes, vasopressors, or both. The need for mechanical ventilation, renal replacement therapy, inotropes, vasodilators, and vasopressors was similar between LVEF groups. Use of immunosuppressive therapies and rates of ECMO-related complications did not differ significantly between groups. Ventricular fibrillation occurred more commonly in the group with an LVEF < 50%, whereas rates of ventricular tachycardia and complete heart block were similar between groups.

Although pre-ECMO LVEF did not differ between groups, patients discharged with an LVEF ≥ 50% demonstrated greater recovery prior to ECMO decannulation, compared with those with an LVEF < 50%. Among patients who died during the index hospitalization (n = 152), 10 had a documented LVEF ≥ 50% at some point prior to death. Of these, 8 had an LVEF ≥ 50% prior to ECMO decannulation, and 3 after decannulation, with one patient represented in both groups. Causes of death in this subgroup were predominantly multi-organ failure (n = 5), followed by neurologic causes (n = 2), mechanical complications (n = 1), cardiac arrest (n = 1), and one unspecified cause.

### Association between LVEF and outcomes

**Figure 2:**
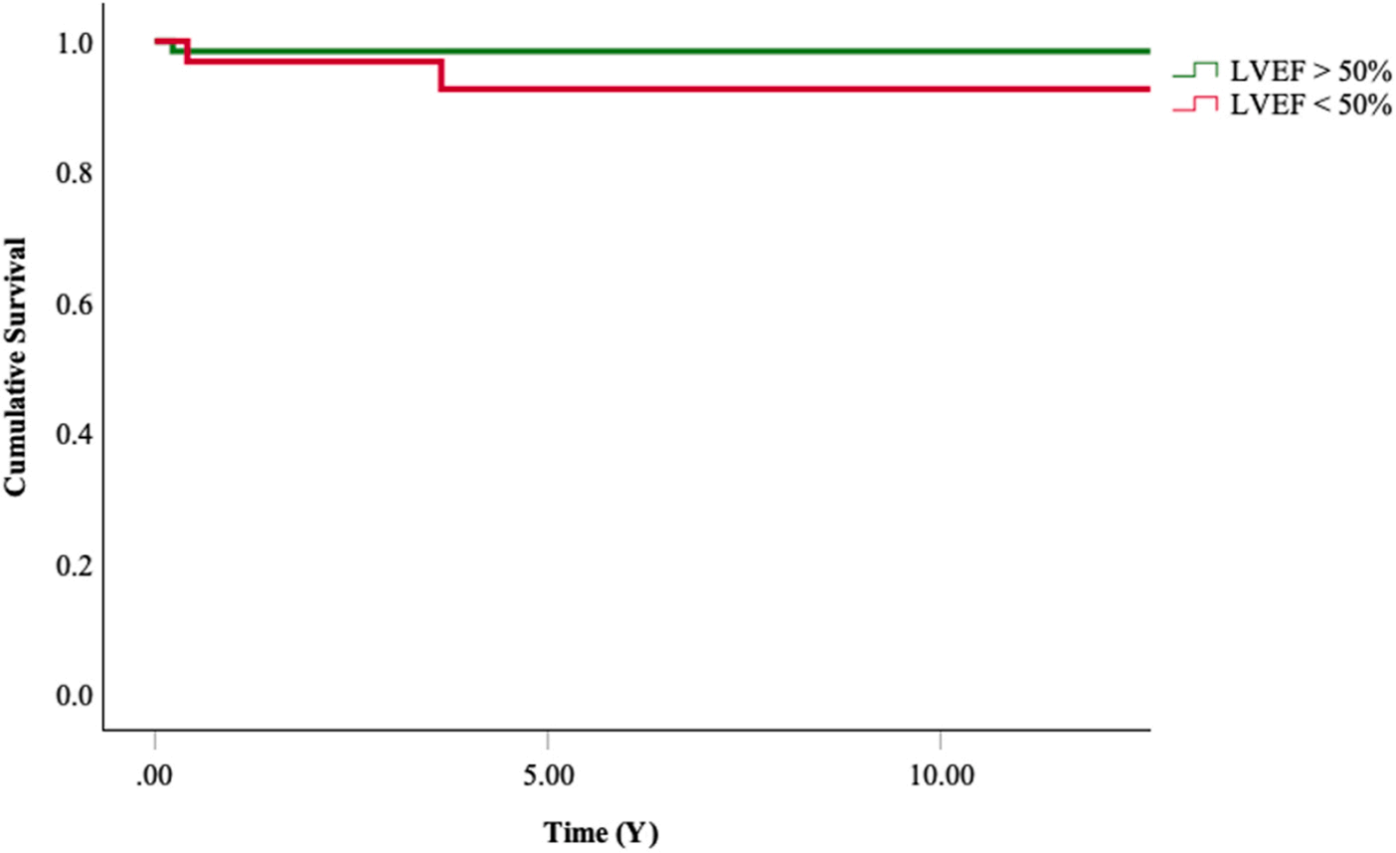
Kaplan-Meier survival curve stratified by left ventricular ejection fraction (LVEF) at time of hospital discharge. This Kaplan-Meier curve illustrates long-term survival among fulminant myocarditis patients requiring venoarterial extracorporeal membrane oxygenation, stratified by LVEF at the time of hospital discharge (< 50% vs ≥ 50%). No significant difference in cumulative survival was observed between the 2 groups during long-term follow-up. These findings suggest that hospital discharge may be a key prognostic milestone in this patient population.

During a median follow-up of 4.4 years (25th-75th percentiles: 1.6-7.9), 2 patients died, 1 had LVAD implantation, and no patients underwent an HTx. By univariate analysis, an LVEF < 50% at discharge (n = 37, 1 death, 1 LVAD), in comparison to an LVEF > 50% (n = 69, 1 death), was associated with a numerically higher risk of the composite endpoint (HR 2.9, 95% CI 0.19-42.9; Fig. 2), although this difference did not reach the level of statistical significance. At 1 year, the risk of death, LVAD, or HTx in patients with an LVEF < 50% was 3%, compared to 2% in those with an LVEF > 50%. Similarly, at 5 years, the composite event rate was numerically higher in patients with an LVEF < 50% (7%), compared to the rate in those with an LVEF ≥ 50% (2%), although these differences were not statistically significant.

### Patient characteristics meeting the composite endpoint

Among patients discharged following VA-ECMO support for FM, 3 subsequently experienced a composite outcome of death or LVAD implantation. The patient characteristics are described in Table 3. The median age of this subgroup was 36 years, with 2 male patients. All 3 had received vasopressors and inotropes during their initial hospitalization, and 2 required mechanical ventilation. One patient underwent renal replacement therapy. Patient 1 was diagnosed with giant cell myocarditis based on biopsy findings and received a durable LVAD 151 days postdischarge. Patient 2 tested positive for parvovirus B19 without histologic inflammation and died 83 days postdischarge. Patient 3 did not undergo biopsy and died 1331 days postdischarge. Immunosuppressive strategies varied: patient 1 received steroids pre-ECMO and mycophenolate mofetil post-ECMO; patient 2 received steroids and IVIG post-ECMO; and patient 3 did not receive steroids or IVIG.

**Table 3:** Patient characteristics postdischarge requiring left ventricular assist device or heart transplantation, or resulting in death

Patient	Age, y	Sex	Biopsy findings	LVEF pre-discharge	CPR	Mechanical Ventilation	RRT	Therapies	Vasopressors	Inotropes	Outcome	Time to composite outcome, d
**1**	25	F	Fulminant destructive eosinophil myocarditis with presence of giant cells	25%				Steroids given pre-ECMO IVIG not given MMF given post-ECMO	√	√	LVAD	151
**2**	40	M	Histologically focal bleeding and areas with vacuolated myocytes and edema. Small foci of damaged myocytes but no inflammation. No eosinophils or giant cells. Positive for parvovirus B19.	57.5%	√	√		Steroids given post-ECMO IVIG given post-ECMO	√	√	Death due to neurologic complication	83
**3**	36	M	N/A	38%	√	√	√	No steroids or IVIG	√	√	Death (unknown cause)	1331

CPR, cardiopulmonary resuscitation; ECMO, extracorporeal membrane oxygenation; F, female; IVIG, intravenous immunoglobulin; LVAD, left ventricular assist device; LVEF, left ventricular ejection fraction; M, male; MMF, mycophenolate mofetil; N/A, not applicable; RRT, renal replacement therapy.

## Discussion

FM is a morbid and deadly disease presenting with cardiogenic shock. In severe cases, VA-ECMO has been shown to be a life-saving therapy. Our findings suggest that the long-term mortality incidence or need for advanced therapy among patients with FM requiring VA-ECMO who survive to hospital discharge is low. In addition, we did not find that LVEF at discharge was associated with an increased long-term risk of the composite outcome. An important point to note is that the very low number of post-discharge events limits the ability of this study to evaluate the prognostic significance of discharge LVEF, and our findings should be interpreted as descriptive rather than inferential.

### Relation to previous work

Only a limited number of studies have described the benefits of VA-ECMO in patients with FM. Among patients decannulated from VA-ECMO, our study described improvement in LVEF prior to decannulation. Early LVEF recovery may serve as a positive prognostic marker in this patient population. The largest cohort study from the Japanese FM registry to date evaluated outcomes in patients with biopsy-proven FM and found that although most patients demonstrated recovery in LVEF by 1 year, 14% continued to exhibit reduced LVEF (< 50%), and this subgroup was associated with significantly higher 1-year mortality or HTx (4% compared to 1% in patients with an LVEF > 50%). An LVEF threshold of 50% was selected a priori in our study to enable comparison with this prior literature. Notably, this study included only a minority of patients supported with VA-ECMO, and all patients were from the Japanese FM registry.

Our international, multicentre study complements the available evidence by focusing exclusively on FM patients who required VA-ECMO support and survived to hospital discharge. In this selective high-risk population, the overall mortality incidence or need for advanced heart therapies was low and was comparable to the risk reported in a wider-risk range of patients hospitalized with myocarditis, both that requiring and that not requiring MCS. Although discharge LVEF < 50% was associated with a numerically higher HR, the limited number of post-discharge events resulted in wide confidence intervals and precluded a statistically definitive association. These findings are directionally consistent with prior registry data, including the Japanese FM registry, but they should be interpreted as underpowered rather than as evidence of an absence of association. Among a high-risk, ECMO-supported population, successful weaning and discharge may represent a key prognostic milestone regardless of residual left ventricular dysfunction. Together, these studies contribute complementary insights into the prognostic implications of LVEF at discharge, with our findings helping to refine risk stratification specifically within the ECMO-treated FM population.

The clinical trajectories of the three patients who reached the composite outcome highlight the long-term risks faced by survivors of FM requiring VA-ECMO. Despite successful hospital discharge, these patients experienced clinically significant adverse events during follow-up, including death or need for durable mechanical support. These findings underscore the fact that even in patients who appear to be clinically recovered, delayed deterioration remains possible. In addition, the heterogeneity in immunosuppressive use and histologic findings reflects the complexity of FM pathogenesis and treatment, reinforcing the need for close monitoring and individualized management strategies.

Some studies have reported long-term outcomes in other etiologies of cardiogenic shock (CS) requiring VA-ECMO. For example, in a retrospective single-centre cohort of 288 VA-ECMO patients with mixed etiology of CS,^8^ the estimated 5-year survival rate was 73% in patients who survived at least 30 days after initiation of VA-ECMO. Only 2% subsequently required durable LVAD support. Finally, a prospective multicentre study evaluated long-term outcomes and disability in 389 patients supported with VA-ECMO, with diverse etiologies of CS, who were discharged from the hospital. The average 1-year mortality incidence was 56.3%, with differences according to etiology of CS. The mortality incidence at 1 year was 54.6% for those requiring perioperative support (n = 108), 65.1% for those with acute myocardial infarction (n = 83), and 57.9% for those with acute decompensated heart failure (n = 76).^9^ Compared with these cohorts, our study demonstrated substantially a lower long-term mortality rate among FM patients who survived to hospital discharge, with comparable rates of LVAD and HTx. These findings suggest that FM-related CS may confer a more favourable long-term prognosis than other etiologies requiring VA-ECMO support.

### Implications for research and clinical practice

LVEF is an accessible and routinely measured parameter, but it represents a simplified marker of myocardial recovery. Prior studies have demonstrated the prognostic importance of cardiac magnetic resonance (CMR) imaging, including assessment of myocardial fibrosis and edema, as well as genetic testing in selected patients with myocarditis.^10^^,^^11^ However, systematic CMR imaging and genotyping were not available across all participating centres in this retrospective international cohort.

Our findings suggest that LVEF at hospital discharge may not be a sufficient standalone marker for long-term risk stratification in FM patients post-VA-ECMO. Given the variable pathology and outcomes—even in patients with preserved LVEF—ongoing clinical surveillance and personalized follow-up strategies are warranted. Additional studies are needed to determine whether integration of biopsy findings, imaging, or biomarkers can improve risk prediction in this population. Future studies should explore whether specific postdischarge management strategies (ie, targeted surveillance or medical therapy intensification) can improve long-term outcomes further in this high-risk group identified based on another clinical characteristic and not just LVEF.

### Study strengths and limitations

This study is the largest of individual patient data of FM survivors post-VA-ECMO to date, and it is the first international multicentre cohort to exclusively assess this population. A major strength is the inclusion of patients from 11 centres across multiple countries, enhancing the generalizability of the findings. The long median follow-up duration of 4.4 years allowed for robust assessment of delayed adverse outcomes. Limitations include the retrospective design and the potential for missing or incomplete data, particularly for postdischarge therapies and outpatient follow-up care. This study is inherently subject to survival bias, as only patients who survived to hospital discharge were included, representing a highly selected population that has already passed the highest-risk phase of illness. Several patients were excluded due to missing discharge LVEF measurements. Comparison of patients with and without available LVEF measurements demonstrated differences in management characteristics, suggesting that these groups may not be directly comparable. This lack of comparability introduces the potential for selection bias, as patients with available LVEF measurement may represent a subset with greater clinical stability, more intensive monitoring, or longer survival during hospitalization. In critically ill patients supported with VA-ECMO, absence of documented discharge LVEF may reflect early death, transfer to another institution, or incomplete echocardiographic assessment during prolonged critical illness, particularly in older cohorts. Patients with no post-discharge follow-up data were excluded, representing a substantial proportion of the original cohort, which may impact the reliability and generalizability of long-term outcome estimates. Although major outcomes, such as death, durable LVAD implantation, or HTx are typically associated with rehospitalization or referral to tertiary centres, the possibility of missed events cannot be excluded completely.

Diagnostic approaches for myocarditis, including the use of endomyocardial biopsy and CMR imaging, were not standardized across participating centres. As a result, the cohort included both biopsy-proven and clinically diagnosed cases, reflecting real-world practice but introducing potential diagnostic heterogeneity. Biopsy results and immunosuppression practices were not standardized across centres, and the decision to perform endomyocardial biopsy was centre-dependent. In addition, we included patients with a variety of etiologies for myocarditis, with some having more aggressive forms of the disease (ie, giant cell myocarditis), which have a poorer prognosis overall.

This analysis was restricted to patients who survived to hospital discharge and therefore, should not be extrapolated to earlier stages of illness, including during ECMO support or prior to discharge. Finally, the small number of postdischarge outcome events limited statistical power and precluded multivariable modelling, thereby precluding meaningful inference regarding the prognostic value of discharge LVEF. Findings should be interpreted as descriptive rather than definitive.

## Conclusion

Among patients with FM requiring VA-ECMO who survived to hospital discharge, long-term mortality and need for advanced heart failure therapies were low. Given limited statistical power, no definitive association between discharge LVEF and post-discharge outcomes could be established. However, hospital discharge may be a favourable prognostic milestone in this population.

## Ethics Statement

This work was approved by the respective ethics board at each participating hospital site. The research reported in this paper adhered to STROBE guidelines.

## Patient Consent

The authors confirm that patient consent is not applicable to this article. This is a retrospective study using de-identified data; therefore, institutional review board approval did not require patient consent.

## Funding Sources

A.C.A. reports receiving funding from the 10.13039/100004411Heart and Stroke Foundation of Canada Clinician-Scientist Program. The other authors have no funding sources to declare.

## Disclosures

The authors have no conflicts of interest to disclose.
