# Supplementary material for: Long-Term Outcomes in Survivors of Fulminant Myocarditis Supported with Venoarterial Extracorporeal Membrane Oxygenation by Discharge Cardiac Function
Source: CJC Open. 2026 Apr 8;8(8):1019–26. doi: 10.1016/j.cjco.2026.03.020 (PMC13480136; doi:10.1016/j.cjco.2026.03.020)
Supplement: Supplementary file 1 [file mmc1.docx]

**Supplemental Table S1.** Baseline characteristics, management strategies, and complications among patients with fulminant myocarditis who survived to hospital discharge, stratified by availability of pre-discharge left ventricular ejection fraction (LVEF).

| Variable | LVEF Available  (N =106) | Missing LVEF  (N=58) | P-value |
| --- | --- | --- | --- |
| Demographics & Clinical Variables |  |  |  |
| Age | 43.0 [29.2–56.0] | 43.4 [27.1–53.8] | 0.812 |
| BMI (m^2^/kg) | 23.0 [20.5–26.2] | 23.6 [21.3–25.1] | 0.806 |
| Male | 61 (45.5%) | 18 (38.3%) | 0.494 |
| Creatinine | 97.3 [76.3–133.3] | 113.2 [69.4–150.6] | 0.620 |
| Management |  |  |  |
| Mechanical Ventilation | 114 (85.1%) | 21 (50.0%) | <0.001 |
| Renal Replacement Therapy | 55 (41.0%) | 20 (42.6%) | 0.865 |
| Immunosuppressive Therapies |  |  |  |
| Steroids | 45 (38.5%) | 6 (23.1%) | 0.008 |
| Immunosuppressive | 8 (8.6%) | 0 (0.0%) | 0.114 |
| IVIG | 33 (28.7%) | 3 (33.3%) | 0.006 |
| Complications on v–A ecmo |  |  |  |
| Major Bleeding | 39 (32.2%) | 8 (17.4%) | 0.124 |
| Neurologic Complication | 9 (6.9%) | 2 (5.4%) | 0.731 |
| Sepsis | 26 (22.2%) | 12 (25.5%) | 0.408 |
| Limb Ischemia | 11 (8.2%) | 8 (22.2%) | 0.102 |

*Categorical variables are presented as percentages in parentheses. Continuous variables are represented as the median with the first and third quartiles in square brackets. BMI: Body Mass Index; ECMO: extracorporeal membrane oxygenation; IVIG: Intravenous Immunoglobulin; LVEF: Left ventricular ejection fraction.*
